# Reconsideration of the Effects of Age on Proximal Femur Structure: Implications for Joint Replacement and Hip Fracture

**DOI:** 10.1371/journal.pone.0164949

**Published:** 2016-10-24

**Authors:** B. C. C. Khoo, J. K. Brown, R. L. Prince

**Affiliations:** 1 Medical Technology & Physics, Sir Charles Gairdner Hospital, Perth, Western Australia, Australia; 2 School of Physics, University of Western Australia, Perth, Western Australia, Australia; 3 School of Medicine and Pharmacology, University of Western Australia, Perth, Western Australia, Australia; 4 Mindways Software Inc., Austin, Texas, United States of America; 5 Department of Endocrinology & Diabetes, Sir Charles Gairdner Hospital, Perth, Western Australia, Australia; Leeds Beckett University, UNITED KINGDOM

## Abstract

**Objectives:**

In recent years quantitative computed tomography (QCT) has allowed precise non-invasive, three dimensional, in vivo measurement of hip structure in large numbers of individuals. The effects of ageing on proximal femur structure are reported and implications for the prevention of hip prosthesis loosening and hip fracture considered.

**Design, Setting and Participants:**

An observational cross-sectional study of proximal femur QCT in 719 unselected female European descent aged 20 to 89 years recruited from US and Australian populations.

**Main Outcomes Measures:**

QCT scans were obtained using software that separates cortical and cancellous bone by a thresholding technique. Voxel based mineral volume and mass was computed for the integral (external), cancellous and cortical compartments of 1 mm wide sections through the femoral neck (FN), trochanter (TR) and intertrochanter (IT) regions.

**Results:**

Over the adult life span total integral volumes at the FN, TR and IT sites expand linearly by between 18 and 37% at the same time as bone mass decreased by 22 to 25% resulting in massive reductions in true volumetric BMD (vBMD) of 40 to 50%. Cancellous volume expansion was larger at 65 to 79% at the three sites. Between the ages of 65 and 75 the average increase in cancellous volume at the IT site was 3.74 cm^3^ (12.1%). Voxel determined FN cortical volume decreased linearly by 43%, as did cortical bone mass so that vBMD did not change substantially. TR and IT cortical volumes decreased 54 and 28% respectively, small reductions in TR and IT cortical vBMD also occurred.

**Conclusions:**

Large endosteal expansion in the area in which hip replacement stem placement occurs may contribute to loosening. Regarding the propensity to hip fracture, periosteal expansion contributes to increased resistance to bending but cortical thinning contributes to loss of bone to resistance to bending forces. Understanding individual hip structure may contribute to individualisation of risk and subsequent targeting of management using pharmaceutical agents.

## Introduction

Osteoarthritis of the hip and hip fracture are more common in women than men and increase in incidence after midlife [1,2]. Joint replacement requires placement of a femoral stem in the proximal femoral shaft. Implant failure, a late complication of hip arthroplasty, affecting about 5–10% [3] of implants is often associated with aseptic loosening [4]. Variation in cancellous volume and shape are well recognised and as such the internal structure of the proximal femur hip may play an important arthroplasty [5] role in reducing implant loosening. Currently the causation of the problem of stem loosening following joint replacement concentrate on the problem of wear particle induced osteolysis over time affecting the propensity to hip fracture. Despite many careful studies of the changes in local anatomy predisposing to hip fracture it is often considered to be due to loss of bone mass as detected by low dual-energy X-ray absorptiometry (DXA) measured areal bone mineral density (aBMD) [6,7].

By age 20 years physeal growth in bone length has ceased but bone shape continues to be influenced by primary bone formation on periosteal surfaces called modelling and bone resorption and formation on the inner surfaces of bone called remodelling. These processes are known to substantially alter bone shape and size over the life span but are not well recognised in the wider medical community [8].

This study examines the effects of age on proximal hip volume and mass in a large number of women from age 20 to 89 years using a sophisticated quantitative CT based analytical protocol and considers the implications of the findings on causation of hip prosthesis loosening and hip fracture.

## Materials and Methods

### Study population

This study is based on a combination of two European descent female cohorts; a United States of America (US) cohort of 628 community dwelling subjects, not on bone active agents, from eleven centers across three climatically distinct regions of the US, and an elderly cohort of 91 unselected community dwelling female Australians subjects aged between 80 and 89 years who were also not on bone active agents apart from calcium. Subjects provided informed consent with institutional review board oversight. This study was approved by the institutional ethical review committees oversight at all study institutions, including Sir Charles Gairdner Hospital, University of Western Australia and the New England Institutional Review Board for US study sites. Participants provided written consent to participate in this study.

### QCT measurement

Female subjects from the US cohort were scanned by computed tomography (CT) with slice thickness varying between 2.5 and 3.2 mm. Females from the Australian cohort were scanned on the Philips Brilliance 64-slice MDCT (Philips, Andover, MA, USA) in Perth, Western Australia, using 1 mm slice thickness quantitative CT (QCT) scan as previously reported [9]. Variations in CT scanner performance and protocol were characterized using the Mindways QA phantom and QA scans as specified by the QCT software manufacturer. In each study a Mindways Phantom (Mindways Software Inc., Austin, TX, USA) was scanned at the same time as the patient to allow precise calibration of mass. Mineral mass estimates were normalized automatically by CT-scanner specific field-uniformity correction values extracted from this QA process.

**Fig 1:**
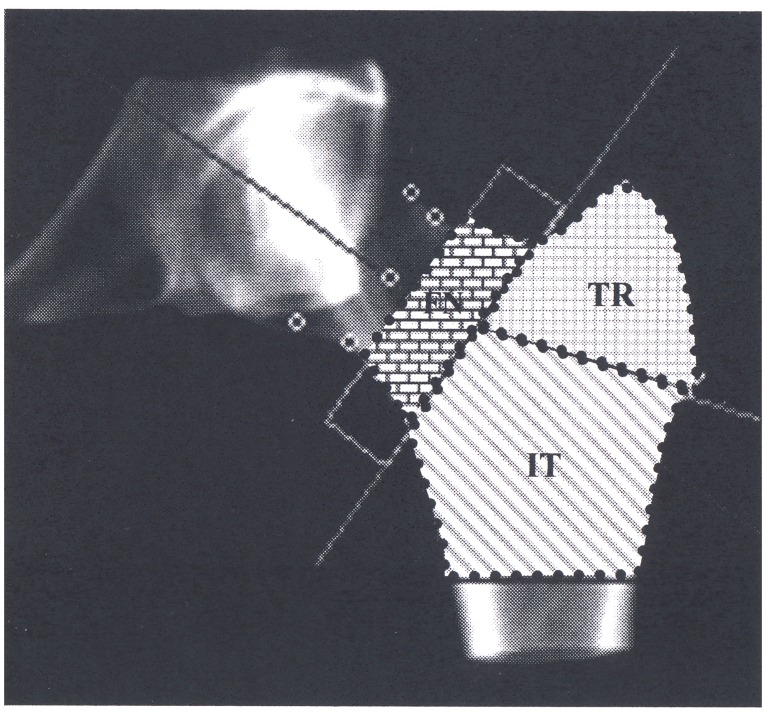
Location of femoral neck (FN), trochanter (TR) and inter-trochanter (IT) ROIs.

The QCT outcome data were analysed using Mindways CTXA Hip software version 4.1 according to the directions provided by the manufacturer (Mindways Software Inc., Austin, TX, USA). Analysis was performed by positioning a region of interest (ROI) centred over the femoral neck as identified on the axial images and a volumetric ROI containing the proximal femur extracted from the CT image data set. To identify the bone surface segmentation of bone from surrounding soft tissue was performed using an adaptive algorithm with a base threshold of 120 mg/cm^3^; this resulted in a data set of voxels identified as bone. This 3D data set of bone voxels was then rotated so that the femoral shaft was vertical in the coronal and sagittal planes with the femoral neck axis in the coronal plane. From the rotated 3D data a 2D image similar to a DXA image was generated by summing all the bone voxels along lines perpendicular to the coronal plane. The lower extent of the inter-trochanter ROI was set at the lower junction of the lesser trochanter and the femoral shaft. The regions of interests (ROI) analyzed included the femoral neck, trochanter and intertrochanter sites determined using an algorithm derived from the DXA approach Khoo et al., 2009 (Fig 1). [9]

From these areas of interest each pixel of the resulting image represented the mass of mineral summed along that line and was further characterized by a known pixel area, and a total volume of bone along the line. Integral bone volume was constrained to be equal to the sum of estimates of cortical and cancellous bone volume. Using a threshold value of 350 mg/cm^3^, integral, cancellous and cortical compartments were identified. Average cortical thickness at the three sites was calculated at age 20 and 89 was calculated as the cubed root of the integral volume minus the cubed root of the cancellous.

The values derived from each patient’s analysis were entered into a database and scatter plots relating the parameter of interest to age modeled for fit using either linear or quadratic outcomes in IBM SPSS statistics Version 20 (IBM, NY, USA) depending on whether the quadratic term achieved statistical significance in the model.

## Results

Fig 1 identifies the location of the femoral neck (FN), trochanter (TR) and intertrochanter sites (IT) sites of the proximal femur. S1 Table shows summary data on the bone volume, bone mineral mass and volumetric bone mineral density at these sites in decades. S1, S2 and S3 Figs show the scatter plots of the bone volume, bone mineral mass and average calculated volumetric bone mineral density for the FN, TR and IT. The modelling of these data with formulae for the lines of best fit are outlined in Table 1.

**Table 1:** Summary of predictive models to bone measures investigated at the FN, TR and IT.

Measure	Fit	P-values	Model equation	% change 20 to 89y^‡^
**Femoral neck (FN)**				
Integral mass (g)	Linear	P<0.001	-0.022Age + 4.711	- 34.8
Integral volume (cm^3^)	Linear	P<0.001	0.027Age + 10.238	+ 17.5
Integral vBMD (gcm^-3^)	Linear	P<0.001	-0.002Age + 0.442	- 43.8
Cortical mass (g)	Linear	P<0.001	-0.018Age + 3.291	- 43.2
Cortical volume (cm^3^)	Linear	P<0.001	-0.030Age + 5.302	- 44.6
Cortical vBMD (gcm^-3^)	Quadratic	P<0.001	-0.00003Age^2^ + 0.003Age + 0.545	+ 1.52
Cancellous mass (g)	Linear	P = 0.008	-0.003Age + 1.418	- 16.7
Cancellous volume (cm^3^)	Linear	P<0.001	0.058Age + 4.936	+ 65.4
Cancellous vBMD (gcm^-3^)	Linear	P<0.001	-0.002Age + 0.245	- 49.9
**Trochanter (TR)**				
Integral mass (g)	Quadratic	P<0.001	-0.0007Age^2^ + 0.058Age + 4.899	-21.9
Integral volume (cm^3^)	Linear	P<0.001	0.125Age + 20.845	+ 36.9
Integral vBMD (gcm^-3^)	Linear	P<0.001	-0.002Age + 0.293	-39.9
Cortical mass (g)	Quadratic	P<0.001	-0.0007Age^2^ + 0.056Age + 1.814	- 53.7
Cortical volume (cm^3^)	Quadratic	P<0.001	-0.0011Age^2^ + 0.086Age + 4.084	- 44.1
Cortical vBMD (gcm^-3^)	Quadratic	P<0.001	-0.00004Age^2^ + 0.003Age + 0.436	- 9.8
Cancellous mass (g)	Linear	P = 0.029	0.002Age + 3.085	+ 5.3
Cancellous volume (cm^3^)	Linear	P<0.001	0.160Age + 13.943	+ 64.5
Cancellous vBMD (gcm^-3^)	Linear	P<0.001	-0.001Age + 0.194	- 34.4
**Intertrochanter (IT)**				
Integral mass (g)	Quadratic	P<0.001	-0.0016Age^2^ + 0.113Age + 13.965	- 27.1
Integral volume (cm^3^)	Linear	P<0.001	0.176Age + 35.403	+ 31.2
Integral vBMD (gcm^-3^)	Quadratic	P<0.001	-0.00004Age^2^ + 0.002Age + 0.380	- 50.1
Cortical mass (g)	Quadratic	P<0.001	-0.0016Age^2^ + 0.125Age + 9.271	- 30.6
Cortical volume (cm^3^)	Quadratic	P<0.001	-0.0015Age^2^ + 0.097Age + 15.112	- 27.7
Cortical vBMD (gcm^-3^)	Quadratic	P<0.001	-0.00004Age^2^ + 0.004Age + 0.605	- 5.4
Cancellous mass (g)	Linear	NS	4.450	0^†^
Cancellous volume (cm^3^)	Linear	P<0.001	0.244Age + 16.437	+ 79.0
Cancellous vBMD (gcm^-3^)	Linear	P<0.001	-0.001Age + 0.224	- 44.6

Model outcomes are categorised into linear or quadratic fits. Linear fits with significant (P<0.05) non-zero slopes are presented with annual rates (/y), together with corresponding P-values in column to the right. Non-significant outcomes are denoted with NS.

^†^: Linear no age effect;

^**‡**^: $\%\text{ change based on modelled fit}=\left(\frac{X_{89}-X_{20}}{X_{20}}\right)$, where X is the bone variable at age 20 or 89.

### Integral results (Table 1, Fig 2)

**Fig 2:**
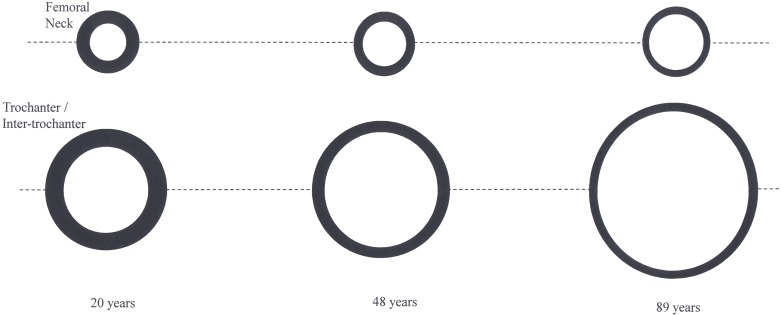
This representation provides a conceptual representation of volume changes occurring at the proximal femur at three time points. The top panel shows the trend for FN volume while bottom panel shows trend for TR volume (which is similar in trend with IT). The area of outer most circles provides an indication of amount of change in integral volume, while the inner circle represents the change in cancellous volume. The shaded region between outer circle and inner circle provides a representation of the change in cortical volume.

Over the age range of 20 to 89 years at each of the three sites large linear increases in the integral volume occurred (FN 17.5%, TR 31.2%, IT 36.9%). FN integral mass decreased linearly by 35% while TR and IT mass was approximately stable until mid-life and subsequently decreased by 22% and 27% respectively with the best fit being quadratic. The effect of the decrease in mass within the bone envelope and the increase in the bone envelope resulted in the true integral vBMD decreasing by between 39.9% and 50.1%. The effects of these age related changes using a theoretical appropriately scaled circular model is presented in Fig 2.

### Cortical compartment results (Table 1, Fig 2)

Between 20 and 89 years, cortical volumes measured by the thresholding technique decreased at the FN, TR and IT by 44.6%, 44.1% and 27.7% respectively. Large cortical mass decreases were also observed FN 43.2%, TR 53.7% and IT 30.6%. The changes in volume and mass were linear from age 20 at the FN but were best described by a quadratic fit at the TR and IT sites due to reduced rate of loss until after mid-life. The combined effect of an increase in external volume and a reduction in cortical volume resulted in a reduction in average calculated cortical width at the FN, TR and IT sites of 56, 53% and 44% respectively. At the FN the calculated volumetric BMD demonstrated a small increase of 1.5% but decreased by 9.8% and 5.4% at the TR and IT sites respectively identifying a reduction in voxel mass in these compartments over the life span.

### Cancellous compartment results (Table 1, Fig 2)

Between the ages of 20 and 89 years, the cancellous compartment expanded linearly by 65.4% at the FN, 64.5% at the TR and 79% at the IT. Between the ages of 65 and 75, an example of the time frame over which hip replacements are expected to survive, the average increase in cancellous volume at the intertrochanter site was 3.74 cm^3^ (12.1%). The cancellous mass changes were variable with a decrease of 17% at the FN, no change at the IT site and an increase of 5% at the TR. Because of the large volumetric increases vBMD decreased substantially FN 50%, TR 34% and IT 45%.

## Discussion

These data identify very large increases in the external (periosteal) and internal (endosteal) dimensions of the proximal femur and major reductions in total bone mass over the life span. The biological processes underlying these changes relate to periosteal bone formation which is active throughout life rather than only being a feature of young adult life, together with the transference of a large amount of endocortical bone from the cortical to the cancellous compartment under the influence of osteoclastic bone resorption on the endocortical surface

The changes observed are larger than, previous studies using dual energy X-ray (DXA) measurement of total hip areal BMD from age 20 that has been reported to fall 27 to 36% [10] often interpreted as implying a reduction in mineral mass within the measured bone region. The increase in external bone size, a major influence on the changes of bone structure with age has been reported dating back to an anthropological study published in 1982 [11]. One aspect of bone volume, projected bone area, has also been measured in vivo using DXA. An NHANES structural geometrical study over the life span has identified a 9% increase in subperiosteal width at the narrow neck and femoral shaft [12, 13, 14]. However the implications of these 2D changes on the magnitude of the true volumetric measures have not been well recognised. Because the femoral neck ROI is constrained by the measurement methodology to a 10 mm length along the femoral neck axis volumetric expansion observed here primarily captures radial expansion in area about the femoral neck axis. Simple geometry confirms that a 9 mm expansion in diameter is consistent with the 18% expansion reported here. A previous estimate using CT based 3D measurement also reported an integral volumetric expansion of 15% [Nicks *et al*.,] [15].

Because of its angular shape the TR ROI is essentially free to expand in three dimensions. A dimensional analysis suggests TR ROI volume should increase as the cube of typical linear dimensions of the TR ROI. Thus the observed 37% volume expansion at the TR ROI is consistent with a linear expansion of approximately 11%.

The distal limit of the IT ROI is constrained in relation to the lesser trochanter which varies in its position along the femur axis based on the judgment of the technician involved. Thus the observed 31% volume expansion at the IT ROI is consistent with an intermediate linear expansion rate estimate bounded between a 9% 3D expansion and a 14% 2D expansion consistent with the estimates of sub-periosteal expansion made from 2D DXA [12]. It should be noted that a lower integral volumetric expansion of 20% at the TR and 23% at the IT sites have been reported by [Nicks *et al*.] [15] perhaps due to differences in the definition of measurement sites.

Although the increase in the volume of the cancellous compartment at the FN best fits a linear process the increase in the rate of expansion at the TR and IT sites best fits a quadratic process associated with increased expansion after mid-life which may represent effects of the loss of estrogen at the menopause causing increased endosteal bone loss. Hip replacement requires excision of the femoral head and placement of a metal stem into the proximal femur cancellous bone. This requires careful consideration on the part of the surgeon to fit the correct size and shape of the stem to the patient’s femur. Given the complexities of achieving this, the failure rate of approximately 10% due to loosening is commendably low. However loosening accounts for at least 40% of all failures and so further understanding of the process is important. The current paradigm is of wear particles increasing bone loss [16]. However the data presented here identify another process that may exacerbate loosening namely that of cancellous expansion over the life of the implant associated with age related changes not necessarily related to wear particle effects. At both the trochanter and inter-trochanter regions, an observed process of simultaneous increases in integral volume with relatively faster increases in cancellous volume, lead to decreases in cortical volume with age (Table 1). This endosteal expansion increases the endosteal cavity, and with age impacts on the fit stability of the hip prosthesis, with the potential of initiating prosthetic loosening and eventual failure [17].

The separation of cortical and cancellous bone compartments are not well captured in two dimensional (2D) projection techniques such as DXA [18] and thus the changes in compartmental volume have not been reliably captured in previous DXA studies. Cortical mass, volume and width at all three sites measured sustain massive reductions that occur over the lifespan. The data on cortical bone mass reduction confirms previous ex vivo studies in a smaller number of individuals concentrating on the proximal FN, the site of intra-capsular fracture identifying the cortex as an important site of bone loss [19, 20, 21]. It is likely that cortical bone loss observed in this and other studies occurs at a rate fast enough to significantly increase the risk of buckling a catastrophic form of localized failure as identified by early studies on effect of bone volume on bone strength [22, 23] and should further focus future research on maintenance of cortical structure for prevention of hip fracture and hip prosthesis loosening.

This study has important limitations; it is a cross sectional study that did not include patients with hip replacements or fracture and so did not determine whether the change in anatomy of the proximal femur observed in these participants occurs in patients with hip disorders. Its strengths are that a substantial number of individuals contributed and reliable positioning of the areas of interest occurred.

In conclusion measuring true 3D anatomy will allow better understanding of hip prosthesis loosening and improved analysis of the resistance to bending and breaking at all proximal hip sites at which fracture is sustained. This new information should further inform research to better calculate individual propensity to fracture and to joint replacement.

### What is already known on this topic?

Femoral neck expansion, bone loss and structural analysis of the elderly have been recognised previously.

### What this study adds

This study now quantifies large expansion and volumetric bone mineral loss at all three important areas of hip disease, the femoral neck, trochanter and inter-trochanter regions across the life span, with implications for loosening of hip implants and reduction in bone structure ability to withstand loads that would have readily been possible at a younger age.

## Supporting Information

S1 Fig Scatter plots for FN (mass, volume, vBMD) corresponding fits. (ZIP)

S2 Fig Scatter plots for TR (mass, volume, vBMD) corresponding fits. (ZIP)

S3 Fig Scatter plots for IT (mass, volume, vBMD) corresponding fits. (ZIP)

S1 Table Bone structure data by decade, site and compartment. (DOCX)
