# Supplementary figures and images for: Reconsideration of the Effects of Age on Proximal Femur Structure: Implications for Joint Replacement and Hip Fracture
Source: PLoS One. 2016 Oct 24;11(10):e0164949. doi: 10.1371/journal.pone.0164949 (PMC5077107; doi:10.1371/journal.pone.0164949)

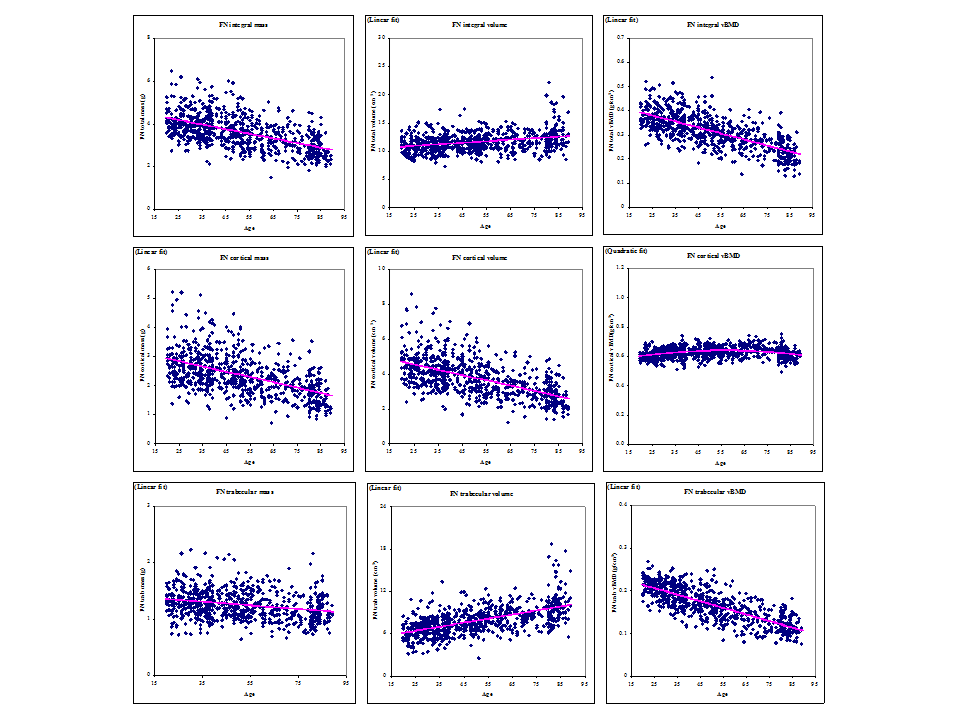

Supplement: S1 Fig — (ZIP) [file pone.0164949.s001.zip › Slide1.TIF]

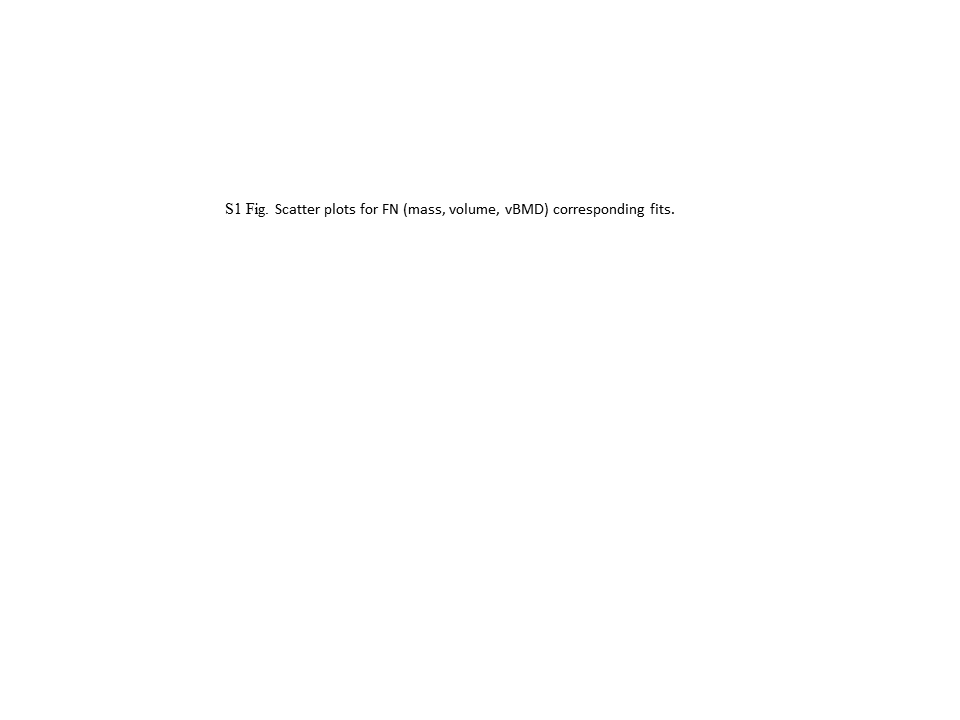

Supplement: S1 Fig — (ZIP) [file pone.0164949.s001.zip › Slide2.TIF]

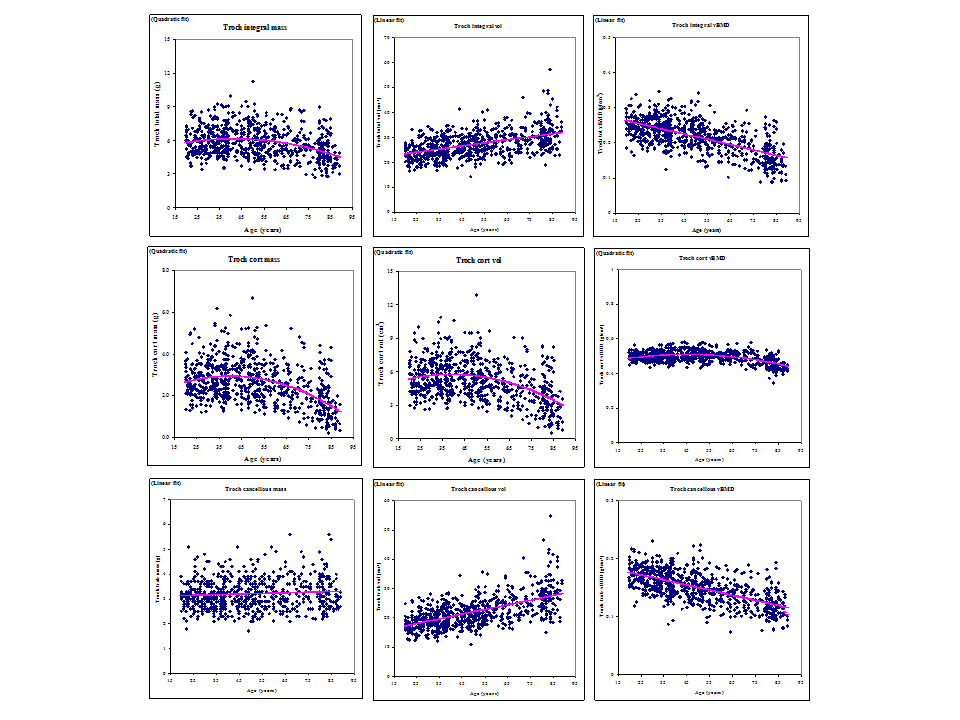

Supplement: S2 Fig — (ZIP) [file pone.0164949.s002.zip › Slide1.TIF]

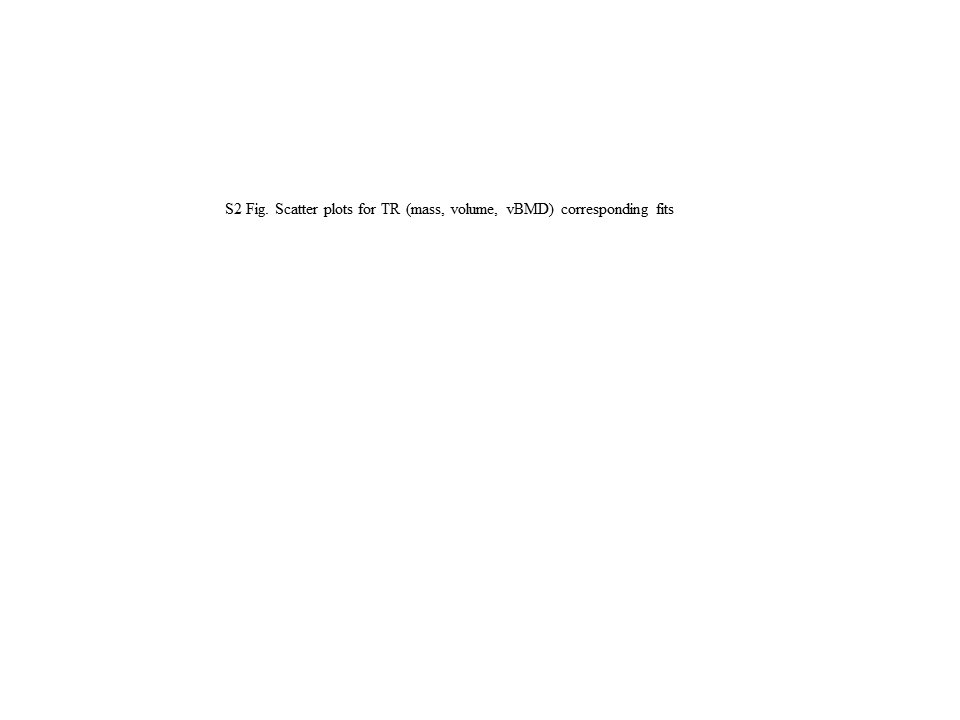

Supplement: S2 Fig — (ZIP) [file pone.0164949.s002.zip › Slide2.TIF]

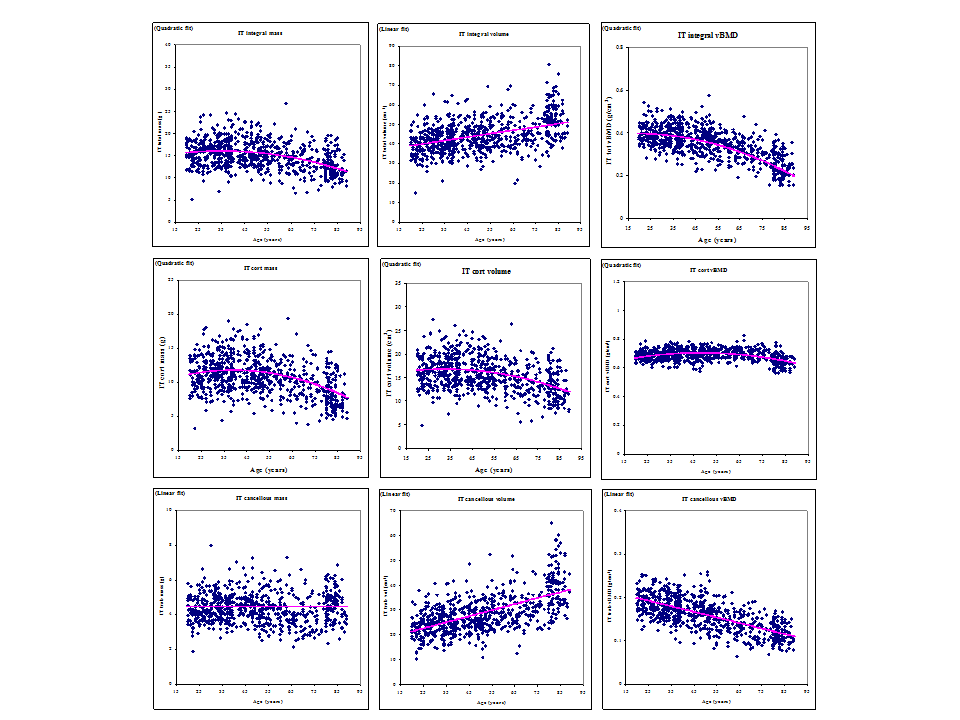

Supplement: S3 Fig — (ZIP) [file pone.0164949.s003.zip › Slide1.TIF]

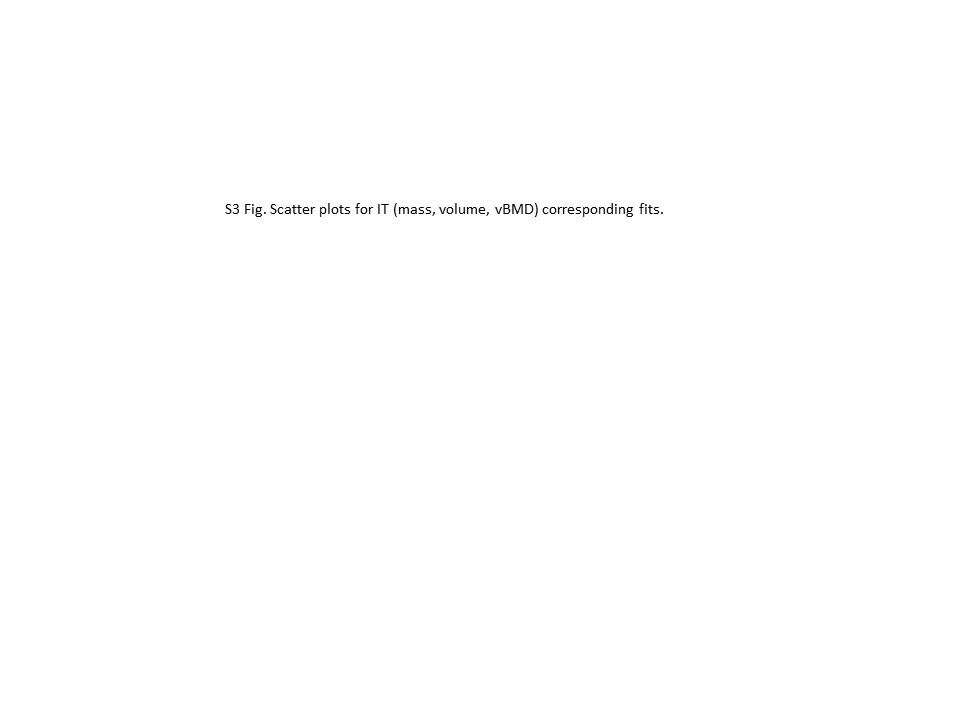

Supplement: S3 Fig — (ZIP) [file pone.0164949.s003.zip › Slide2.TIF]
