# Supplementary material for: Reconsideration of the Effects of Age on Proximal Femur Structure: Implications for Joint Replacement and Hip Fracture
Source: PLoS One. 2016 Oct 24;11(10):e0164949. doi: 10.1371/journal.pone.0164949 (PMC5077107; doi:10.1371/journal.pone.0164949)
Supplement: S1 Table — (DOCX) [file pone.0164949.s004.docx]

| S1 Table. Bone structure data by decade, site and compartment | | | | | | | |
| --- | --- | --- | --- | --- | --- | --- | --- |
| **Age (years)** | **20-29** | **30-39** | **40-49** | **50-59** | **60-69** | **70-79** | **80-89** |
| N | 115 | 171 | 96 | 103 | 70 | 51 | 113 |
| BMI (kgm^-2^) | 24.84 (5.04) | 24.77 (5.18) | 27.82 (7.00) | 27.85 (6.10) | 26.95 (4.98) | 27.05 (5.14) | 26.17 (3.94) |
|  | **Femoral Neck Site** | | | | | | |
| Integral Mass (g) | 4.08 (0.69) | 3.97 (0.65) | 3.86 (0.72) | 3.59 (0.67) | 3.27 (0.63) | 3.11 (0.60) | 2.89 (0.53) |
| Integral Volume (cm^3^) | 10.97 (1.38) | 11.17 (1.47) | 11.59 (1.44) | 11.71 (1.47) | 11.70 (1.66) | 11.79 (1.35) | 12.77 (2.36) |
| Integral vBMD (g cm^-3^) | 0.37 (0.05) | 0.36 (0.05) | 0.33 (0.05) | 0.31 (0.05) | 0.28 (0.05) | 0.26 (0.05) | 0.23 (0.05) |
| Cortical Mass (g) | 2.75 (0.72) | 2.67 (0.64) | 2.56 (0.64) | 2.32 (0.59) | 2.08 (0.52) | 2.00 (0.49) | 1.73 (0.47) |
| Cortical Volume (cm^3^) | 4.51 (1.11) | 4.25 (0.92) | 4.03 (0.96) | 3.64 (0.86) | 3.22 (0.76) | 3.07 (0.71) | 2.82 (0.72) |
| Cortical vBMD (gcm^-3^) | 0.61 (0.03) | 0.63 (0.04) | 0.63 (0.04) | 0.64 (0.03) | 0.64 (0.04) | 0.65 (0.04) | 0.61 (0.04) |
| Cancellous Mass (g) | 1.32 (0.25) | 1.30 (0.24) | 1.30 (0.25) | 1.28 (0.23) | 1.19 (0.25) | 1.11 (0.24) | 1.16 (0.24) |
| Cancellous Volume (cm^3^) | 6.46 (1.45) | 6.92 (1.49) | 7.55 (1.46) | 8.07 (1.47) | 8.48 (1.57) | 8.72 (1.31) | 9.95 (2.48) |
| Cancellous vBMD (gcm^-3^) | 0.21 (0.02) | 0.19 (0.02) | 0.17 (0.03) | 0.16 (0.03) | 0.14 (0.03) | 0.13 (0.03) | 0.12 (0.02) |
|  | **Trochanter** | | | | | | |
| Integral Mass (g) | 5.84 (1.14) | 6.08 (1.21) | 6.19 (1.34) | 6.01 (1.38) | 5.59 (1.32) | 5.41 (1.40) | 4.97 (1.27) |
| Integral Volume (cm^3^) | 23.81 (3.66) | 25.42 (3.83) | 26.32 (3.98) | 27.56 (4.45) | 28.54 (4.74) | 29.75 (4.97) | 31.44 (6.02) |
| Integral vBMD (gcm^-3^) | 0.25 (0.03) | 0.24 (0.04) | 0.24 (0.04) | 0.22 (0.04) | 0.20 (0.03) | 0.18 (0.04) | 0.16 (0.04) |
| Cortical Mass (g) | 2.70 (0.82) | 2.90 (0.91) | 3.01 (0.95) | 2.77 (0.99) | 2.35 (0.84) | 2.17 (1.05) | 1.67 (0.96) |
| Cortical Volume (cm^3^) | 5.40 (1.49) | 5.72 (1.54) | 5.82 (1.61) | 5.42 (1.73) | 4.67 (1.48) | 4.32 (1.85) | 3.58 (1.83) |
| Cortical vBMD (gcm^-3^) | 0.50 (0.02) | 0.50 (0.03) | 0.51 (0.03) | 0.51 (0.03) | 0.50 (0.03) | 0.49 (0.03) | 0.45 (0.03) |
| Cancellous Mass (g) | 3.14 (0.52) | 3.17 (0.53) | 3.18 (0.58) | 3.23 (0.65) | 3.24 (0.68) | 3.24 (0.66) | 3.29 (0.62) |
| Cancellous Volume (cm^3^) | 18.41 (3.13) | 19.70 (3.47) | 20.49 (3.43) | 22.14 (4.01) | 23.88 (4.21) | 25.42 (4.92) | 27.87 (6.26) |
| Cancellous vBMD (gcm^-3^) | 0.17 (0.02) | 0.16 (0.02) | 0.16 (0.02) | 0.15 (0.02) | 0.14 (0.02) | 0.13 (0.02) | 0.12 (0.02) |
|  | **Intertrochanter** | | | | | | |
| Integral Mass (g) | 15.59 (2.95) | 16.09 (2.90) | 16.32 (3.19) | 15.51 (2.80) | 14.28 (3.25) | 13.52 (3.04) | 12.92 (2.84) |
| Integral Volume (cm^3^) | 39.69 (6.89) | 42.07 (6.85) | 44.09 (6.54) | 43.71 (7.01) | 44.62 (9.21) | 45.51 (6.64) | 52.23 (8.86) |
| Integral vBMD (gcm^-3^) | 0.39 (0.05) | 0.38 (0.05) | 0.37 (0.06) | 0.36 (0.06) | 0.32 (0.05) | 0.30 (0.05) | 0.25 (0.06) |
| Cortical Mass (g) | 11.25 (2.51) | 11.63 (2.49) | 11.74 (2.71) | 11.25 (2.25) | 10.18 (2.56) | 9.59 (2.55) | 8.39 (2.47) |
| Cortical Volume (cm^3^) | 16.33 (3.39) | 16.75 (3.18) | 16.78 (3.46) | 15.88 (2.96) | 14.35 (3.29) | 13.65 (3.23) | 12.92 (3.30) |
| Cortical vBMD (gcm^-3^) | 0.69 (0.03) | 0.69 (0.04) | 0.70 (0.04) | 0.71 (0.03) | 0.71 (0.04) | 0.70 (0.04) | 0.64 (0.04) |
| Cancellous Mass (g) | 4.34 (0.77) | 4.45 (0.75) | 4.57 (0.81) | 4.25 (0.86) | 4.10 (0.99) | 3.92 (0.89) | 4.53 (0.90) |
| Cancellous Volume (cm^3^) | 23.36 (5.06) | 25.32 (5.40) | 27.31 (5.56) | 27.84 (5.86) | 30.27 (7.16) | 31.86 (5.62) | 39.31 (0.83) |
| Cancellous vBMD (gcm^-3^) | 0.19 (0.03) | 0.18 (0.03) | 0.17 (0.03) | 0.16 (0.03) | 0.14 (0.03) | 0.13 (0.03) | 0.12 (0.02) |
| Results are mean (SD) | | | | | | | |
